# Zooming Versus Slacking: Videoconferencing, Instant Messaging, and Work-from-Home Intentions in the Early Pandemic

**DOI:** 10.1177/00027642231155364

**Published:** 2023-03-09

**Authors:** Jeremy Schulz, Øyvind Wiborg, Laura Robinson

**Affiliations:** 1 Researcher, Institute for the Study of Societal Issues, UC Berkeley, Berkeley, CA, USA; 2 Department of Sociology and Human Geography, University of Oslo, Oslo, Norway; 3 Department of Sociology, Santa Clara University, Santa Clara, CA, USA

**Keywords:** remote work, communication modalities, Zoom, Slack, technology

## Abstract

This article explores key determinants of the intention to work from home (WFH) among U.S. adults in the early phase of the pandemic. Leveraging nationally representative survey data collected in the initial stages of the pandemic, it explores the role of modalities of communication alongside the more frequently studied behavioral, occupational, and sociodemographic factors in shaping WFH intentions as reported by survey respondents. Venturing beyond prior studies of remote work and remote work intentions, the study finds that the frequency of text messaging platform (e.g., Slack) usage and the frequency of videoconferencing (e.g., Zoom) exhibit diametrically opposed effects on the intentions to WFH in the future. Whereas a higher frequency of text messaging platform usage is linked to a preference for more intensive future WFH, a higher frequency of videoconferencing platform usage is associated with the opposite preference. Additionally, the effect of the intensity of respondents’ engagement with these two communication modalities on their intentions is mediated by pre-pandemic WFH experience as well as the intensity of interruptions in their WFH environment. Intensive videoconferencers (Zoomers) who work in high-interruption environments are particularly averse to future WFH. Conversely, intensive messagers (Slackers) who work from home substantially prior to the pandemic report express a preference for more frequent WFH in the future.

## Introduction

This study investigates how two primary digital communication modalities—instant text messaging and videoconferencing—shaped intentions to work-from-home (WFH) in the future during the COVID-19 pandemic. The post-pandemic spread of working from home has attracted the attention of social science from a variety of angles and analytic traditions. As far as definitional characteristics of WFH are concerned, overviews of working from home note the distinction between formalized WFH arrangements—typically decided consensually between workers and their employers—and informal WFH practices, which can often take place alongside and in addition to more traditional work-from-jobsite practices (Mas & Pallais, 2020). Another important distinction can be drawn between “fully remote” work and work which is done partially from home and partially in another location. However WFH is characterized and measured, its prevalence has undoubtedly increased on account of the pandemic. Measured in terms of workdays, survey-based self-report studies of U.S. workers have suggested a dramatic pandemic-driven expansion of working from home from roughly 5% of all workdays before the pandemic to roughly 20% of all workdays during the first year of the pandemic (Barrero et al., 2021). Moreover, at the peak of the pandemic lockdowns in the United States, in May 2020, an unprecedented 40% of working-age persons were working from home. Somewhat unexpectedly, these studies have indicated that, even after the pandemic receded, the large-scale diffusion of WFH practices will persist. A survey of U.S.-based workers estimates that, although few employers are planning to permit their workforces to work remotely all the time, the median U.S. worker is still expecting to be able to spend between one-fifth and one-quarter of all paid workdays at home as opposed to the office or worksite (Bloom et al., 2022, Barrero et al., 2021, p. 2).

During the pandemic, WFH practices varied strongly along many of the axes which divide the workforce into more and less advantaged groups. For instance, the proportion of workers who worked from home during the pandemic lockdowns reflected both education and income gradients (Barrero et al., 2021). However, new lines of cleavage appeared as well between those workers who worked in more “teleworkable” jobs where most tasks could be done remotely as compared to those who worked in non-teleworkable jobs which involved necessary physical interaction with objects or face-to-face interaction with clients, co-workers, and customers (Dey et al., 2020; Dingel & Nieman, 2020). At the same time, workers in more congested urban areas, often facing more taxing commutes, also tend to WFH more than their peers in less congested areas. Since the onset of the pandemic, a persistent mismatch between the WFH preferences of workers—particularly office workers—and the plans of employers has surfaced. Workers appear to consistently desire more WFH time than employers want to allow (Barrero et al., 2021).

Where predicting the intention to WFH is concerned, research initiated during the pandemic remains surprisingly sparse. One study of U.S. employers and workers concludes that preferences for WFH arrangements among organizational employers appear to vary according to the size, industry, and earning level of the organization, with larger and more profitable employers tending to incorporate more WFH time into their post-pandemic plans (Barrero et al., 2021). According to this survey-based study, preferences among workers for WFH arrangements at the individual level reflect gender-specific patterns as well as educational level and the overall quality of internet connectivity (Barrero et al., 2021). Such studies provide convincing evidence that a broad range of life course and demographic factors can influence the frequency with which individuals WFH.

The study which comes closest to attempting an explanation of WFH preferences focuses on working residents of the Australian city of Melbourne—a city that experienced prolonged and repeated lockdowns during the pandemic (Jain et al., 2022). This study establishes that both sociodemographics and psychosocial attributes played roles in accounting for variation across individuals in terms of WFH intentions within this relatively homogenous group. For instance, attitudes and norms regarding work-life balance and “perceived control” over work conditions substantially shaped WFH intentions in the early stages of the pandemic among the members of this Australian group (Jain et al., 2022). At the same time, sociological research has revealed that the degree of experienced work-life conflict also bears on workers’ orientations to working from home: those workers who experience more work-life conflict—often parenting workers who are caring for school-age children (Schieman et al., 2021)—will most likely find working from home less satisfying than those who do not have parental obligations. Since childcare and housework tasks are most often carried out by women, even when they belong to remote-working couples (Dunatchik et al., 2021), such work-life conflict will most likely make its presence felt among working mothers, thereby reducing the appeal of working from home for this group relative to other sociodemographic groups (Fan & Moen, 2021).

Despite the interest in tracing the sources of WFH preferences and intentions, no extant study examines specific communication modalities as potential independent determinants of WFH intentions, either presently or during the early stages of the pandemic. We remedy this oversight by exploring the specific associations between WFH intentions and two of the most commonly used digital communications modalities—videoconferencing and text messaging—among U.S. adults in the early stages of the pandemic. This study addresses this question empirically by drawing on a nationally representative survey of U.S. adults administered in the fall of 2020, roughly 8 months into the pandemic, to ascertain how the intensity of two key modalities of communication impact preferences vis-à-vis future WFH.

It is well worth investigating potential linkages between these communication modalities and WFH intentions. Numerous studies carried out before and during the pandemic point to the important consequences of videconferencing platforms (e.g., Zoom) and instant messaging platforms (e.g., Slack) for work satisfaction and stress levels at work (Cameron & Webster, 2005; Darics, 2014). Studies in communication, sociology, and social psychology conducted after the beginning of the pandemic hint at the possibility that modalities of communication might influence individuals’ orientation toward working from home in the same way they impact psychological characteristics such as experiencing stress. Conceptually these impacts can be framed as the behavioral and subjective outcomes of the technological “affordances” (Davis, 2020) associated with specific communication modalities which facilitate and inhibit differing kinds of communication behaviors as well as different stances toward the channels and WFH as a whole. In this study, we, therefore, attempt to better understand the associations between these two communication modalities and WFH intentions, accounting for multiple relevant differences across individuals along other relevant dimensions of variation.

## Background: Research on Communication Modalities and Work

Existing research on communication modalities focuses on the impacts of such communication technologies on productivity, stress, and mental health. For instance, research has uncovered evidence that the extensive use of chat messaging platforms such as Slack, Microsoft Teams, and Google Workspace can induce “communication overload” (Molla, 2019), eroding workers’ focus and attention, exacerbating stress, and reducing overall productivity. On the other hand, many workers prefer mobile and computer-based instant messaging to phone conversations, particularly when the topic of the exchange is relatively superficial and/or it involves more than two communicative parties (Lebbon & Sigurjónsson, 2016).

Although the potential links between communication modalities and WFH intentions have not yet drawn attention from empirical social science, communication modalities have been tied to stress in ways which suggest some potential connections with WFH intentions. A recent American post-pandemic analysis of the links between perceived stress and the five main communication modalities of email, phone calls, texting, video chat, and instant messaging, all modalities which are heavily utilized in remote work, found that only texting and videoconferencing exacerbated stress among workers during the covid lockdown period (Pennington et al., 2022). Emails and phone calls did not have similarly statistically discernable effects on stress levels.

Ties between excessive videoconferencing and stress and fatigue in particular have been well established in pre-pandemic literature. While videoconferencing platforms perform better at reproducing more of the cues transmitted in face-to-face communication than text-based platforms and hence serve better for particular communication tasks (Standaert et al., 2016), they can also induce more stress when used in excess on account of cognitive overload. The heavy demands imposed on participants’ attention (Bailenson, 2021) by videoconferencing, as a “multimodal” form of synchronous audiovisual communication can result in what is colloquially known as “Zoom fatigue.” In theory such fatigue is less likely to afflict participants who are relying on workplace chat or messaging programs which fall closer to the unimodal or “lean” end of the media richness spectrum (Koke, 2004).

IM systems which lie on the “leaner” end of the media richness spectrum offer certain advantages over videoconferencing, inasmuch as they generate a “persistent” record of ongoing communications in an accessible form and a sense of continuous copresence for geographically dispersed co-working communicators (Darics, 2014). Instant messaging (IM), as opposed to videoconferencing, can be regarded as either fully synchronous, “quasi-synchronous,” or fully asynchronous, depending on how it is used in a specific context (Darics, 2014). Though text messaging platforms can be employed in ways that replicate the timing and flow of real-time interaction, they nevertheless can also be utilized in ways akin to email, with long delays between replies. Research into the use of IM systems in work contexts has established that such systems are often used in tandem with other digital and non-digital communication modalities and that using IM systems signals “informality” (Cameron & Webster, 2005). Viewed from a “social presence” perspective, as compared with videoconferencing, IM is a “low-presence” modality useful for what many would consider low-complexity tasks (Pazos et al., 2013). The “presence awareness” features of IM systems make it possible to initiate communication in ways that minimize disruptions for either the initiator or receiver by signaling communication availability continuously. Finally, and perhaps most significantly, pre-pandemic research suggests that workers tend to rely on IM systems for those communications which are perceived as less likely to lead to conflict or dissensus between them and co-workers or managers (Pazos et al., 2013).

Despite their ubiquity, research into WFH practices and intentions has not yet sought to uncover links between specific communication modalities, technology affordances (Davis, 2020), and WFH intentions. In this study, we inquire into whether communication modalities have any discernable consequences for workers’ intentions to WFH in the future. We, therefore, set out to see whether the frequency of either text messaging or videoconferencing has any consequences for individuals’ intentions to WFH in the future.

## Research Questions and Hypotheses

Our primary research question can be formulated in the following way: How did the intensity or frequency of employing digital communication modalities, namely instant messaging and videoconferencing, affect WFH intentions as indexed by individuals’ preferred frequency of future WFH? Building on this primary research question, we investigate two related secondary research questions. These questions are relevant to the potential confounding and mediating impacts of two other background factors—pre-pandemic WFH experience and the attentional environment—vis-à-vis the associations between the two communication modalities and the outcome. We therefore additionally ask the following two corollary questions: (a) To what extent does pre-pandemic familiarity with WFH confound any associations between communication modalities and WFH intentions and (b) to what extent does the attentional environment of the individual—as operationalized by the frequency of interruptions—mediate or confound such associations?

## Data Source: Wave 77 of the Pew American Trends Panel

This study addresses the primary and secondary questions through an analysis of cross-sectional survey data provided by Wave 77 of the Pew Research Center’s American Trends Panel (ATP) initiated in 2014. The ATP is designed as a nationally representative survey of U.S. adults. Wave 77 of the ATP survey consists of 10,332 respondents recruited through a random sampling of residential addresses throughout the United States, generating a response rate of 88% (Pew Research Center, 2021). Wave 77 of the panel was carried out between October 13 and October 19, 2020, roughly 6 months into the COVID-19 pandemic. Though most surveys were administered online, where panelists did not have internet access, Pew provided them with a tablet and a wireless internet connection so that they could complete the survey. For Wave 77, a sampling design is paired with a multistep weighting scheme reflecting multiple dimensions of the surveyed population.

## Dependent Variable: Future WFH Preferences

To measure preferences to WFH in the future, we use a dichotomous outcome. Our outcome measure is based on a distinct ordinal scale based on the following question: “Looking ahead to when the coronavirus outbreak is over, if you had a choice would you want to work from home. . .”. The response categories are ordered (except for the last category), listed as 1 = *all of the time*, 2 = *most of the time*, 3 = *some of the time*, 4 = *rarely*, 5 = *never*, and 6 = *not sure*. Some of the response categories have few observations. And since we were mainly interested in the proportion of wanting to WFH in the future, we have collapsed the values into the two most frequent and important original categories: “All of the time” and “most of the time” are collapsed into one category (coded 1), and the remaining original categories (“some of the time,” “rarely,” and “never/not sure”) into the other category (coded 0).

## Focal Independent Variables: Frequency of Video Chatting and Instant Messaging

We focus on two independent scale items as our focal independent variables. Both outcomes are operationalized through two ordinal scale questions. Respondents were asked, “How often, if ever, do you use the following online tools to keep in touch with co-workers while working from home?” The frequency scale for both items was 1 = *Often*, 2 = *Sometimes*, 3 = *Hardly ever*, and 4 = *Never*.

(1) “Instant messaging platforms such as Slack or Google Chat”
(2) “Video calling or online conferencing services like Zoom or Webex”

These two ordinal measures have only four categories making them unsuitable as a proxy for a linear, continuous predictor variable. And both measures have very few observations on some categories to use a dummy variable for each category in a regression model. We have therefore collapsed the central responses into two categories: “Often” and “sometimes” are coded 1, and “Hardly ever” and “never” are coded 0. At the same time, we are primarily interested in the division between those who have been using versus those who have rarely/never used video chatting and instant messaging (Table 1).

**Table 1.** Summary Statistics for all Variables in Regression Models.

Analytic variable	Unweighted proportion	*SD*
Outcome: preferred WFH frequency		
Some/never/rarely/DK	.41	.49
All/most of the time	.59	.49
Videoconferencing frequency		
VID never/sometimes	.40	.49
VID often	.60	.49
Instant messaging frequency		
IM never/sometimes	.60	.49
IM often	.40	.49
Sociodemographics and Education		
Male	.47	.50
Female	.53	.50
White non-Hispanic	.66	.47
Black non-Hispanic	.09	.29
Hispanic	.16	.36
Other	.03	.18
Asian non-Hispanic	.06	.23
18–29	.12	.32
30–49	.49	.50
50–64	.30	.46
65+	.08	.28
Children in HH	.35	.48
No children HH	.65	.48
College graduate+	.78	.41
Some college	.18	.38
H.S. graduate or less	.04	.19
Occupational Characteristics		
Employee	.72	.45
Supervisor	.28	.45
A private company or business	.50	.50
A nonprofit organization	.11	.32
Government	.24	.43
Self-employed	.12	.33
Other	.02	.13
Employment: full-time	.84	.37
Employment: part-time	.16	.37
Background variable (pre-pandemic WFH)		
Past WFH never/sometimes	.78	.42
Past WFH most of the time	.22	.42
Mediating variable (interruptions)		
Less interruptions	.70	.46
More interruptions	.30	.46
Total non-missing observations	2,437	2,437

*Note*. WFH = work-from-home; VID = videoconferencing.

## Focal Control Variables: Pre-Pandemic WFH and Attentional Environment (Interruptions)

To better grasp the conditioning effect of experiential background factors, the study incorporates a background factor that has been shown to affect WFH intentions, namely pre-pandemic WFH practices (Jain et al., 2022). The study, therefore, operates from the presumption that those who have worked from home before the pandemic approach WFH from a different perspective than those who began working from home as a result of the unforeseen circumstances imposed on them and their employers by the pandemic and governments’ response to the pandemic (Jain et al., 2022, p. 61).

In this study, the variable representing pre-pandemic WFH practices is derived from a frequency scale survey item. Respondents are asked to report how often they worked from home according to the following 5-point frequency scale: all of the time (5), most of the time (4), some of the time (3), rarely (2), or never (1). The unweighted proportion of respondents working from home all the time prior to the pandemic was 6.56% (*n* = 384), while 59% of the unweighted sample (*n* = 3,437) had never worked from home prior to the pandemic. In specifying the analytic variable representing pre-pandemic WFH practices, we again created a dummy variable, splitting the sample between respondents who had rarely or never worked from home before the pandemic (76% of the analytic sample, *n* = 4,449) versus those who had worked all, some, or most of the time from home before the pandemic (24%, *n* = 1,404). We label the former group pre-pandemic WFH minimalists and the latter as pre-pandemic WFH maximalists. We treat this variable as a background variable concerning our focal theoretical predictor variables and our dependent variables, as well as a proxy for the relative remote-friendliness or “teleworkability” (Dingel & Neiman, 2020) of the respondent’s job, employer, and occupation. Both the original forms and the dichotomous specification of this conditioning variable are strongly and positively correlated with our outcome variable in the unweighted sample (Cramér’s *V* = 0.31, logit odds ratio [*OR*] = 5.59 *p* > .001 for the dichotomous specifications).

In constructing the models, we also suppose that the respondents’ attentional environment—namely, the degree to which they can concentrate on their work free from distractions—makes a difference in regard to the implications of instant messaging and videoconferencing for their WFH intentions. To account for the attentional environment as a potential factor mediating or interacting with this association, we turn to a survey item tracking the ease with which respondents can concentrate without interruptions on their work in their WFH environment. This question was posed to the subset of respondents who worked from home at least “some of the time” during the pandemic, which accounted for the vast majority of the non-missing respondents who were asked about their current WFH practices. The survey item was phrased as follows: “Since the beginning of the coronavirus outbreak, how easy or difficult has it been for you to get your work done without interruptions?” and offers the following response categories: 1 = *very easy*, 2 = *somewhat easy*, 3 = *somewhat difficult*, and 4 = *very difficult*.

## Other Control Variables

### Sociodemographic Covariates

Our models adjust for three prominent sociodemographic attributes of the respondents, namely age, gender, and race/ethnicity. All three factors could plausibly have an effect on both the frequency of Information and communication technologies (ICT) use and the intention to WFH in the future.

*Age*: Age has been included in previous studies as a control variable (Jain et al., 2022). We use the original categorical form of the variable with four age groups. The analyses include dummy variables for each of the age groups. The youngest age group is used as the reference category.
*Gender*: Gender has been included in previous studies of WFH practices as a control variable. Males are coded 1 and females are coded 0.
*Race/Ethnicity*: Race/ethnicity has been incorporated into previous studies of ICTs and stress levels as a control variable (Pennington et al., 2022). We use the original categorical form of the variable with five race/ethnicity groups. The analyses include dummy variables for each of the categories, using “White non-Hispanic” as the reference category.
*Presence of school-age children in household* (dummy): This factor has been included in previous studies of remote work as an important control variable (Barrero et al., 2021). Here the reference category is taken as households without resident school-age children.
*Respondent’s Education* (categorical: four levels): Respondent’s education may impact their intentions to WFH, both directly and indirectly, as well as their use of ICTs for work purposes. It has also been included in previous studies as a control variable (Barrero et al., 2021). This variable has been dichotomized with a split between those who have some college education (or less) and those who have a college degree (BA) or more education.

### Occupational and Work Situation Covariates

Our models control for three important features of the respondent’s work/employment situation which have been demonstrated to influence both their use of ICTs for work and their intentions vis-à-vis working from home.

*Respondent’s employment/work status* (full-time, part-time, none): Employment status has been included in previous studies of WFH as a control variable (Jain et al., 2022).
*Supervisory/managerial responsibility* (dummy): Respondents’ managerial rank and responsibilities may impact their intentions to WFH, both directly and indirectly, as well as their use of ICTs for work purposes.
*Employment sector* (unordered categorical): As we know from prior research into WFH practices and intentions, such preferences may vary along with the individuals’ occupation and employment sector. As the Pew survey provides for over 10 distinct occupations, we chose to incorporate employment sector for the sake of parsimony. In the survey respondents’ occupations are sorted into the following five occupational sectors: private, nonprofit, government, self-employment, and other.

## Analytic Strategy and Methods

Our analytical setup builds on the standard control variable framework. In the first set of modeling (1), the models focus on the independent focal variables, incrementally including different sets of control variables. The first strategy rules out the role of likely and observed confounding factors. Obviously, we cannot reach any causal inferences since we are likely to omit other confounding dimensions that are not observed in the data. In the last two sets of modeling (2 and 3), we focus on the interactions between our independent focal variables and the central control variables. In doing so, we carry out separate analyses for the subsamples split by pre-pandemic WFH and interruptions at home to ease the presentation of these interactions. We additionally implement full models with the interaction terms included.

The primary regression results were obtained through several series of survey-weighted binomial logistic regression models estimated with both average marginal effects (AMEs) and OR coefficients. Base models and models with covariates were all implemented with the dichotomous specifications of the dependent variable, the focal independent variables, and the potential mediating variables. Following Hosmer and Lemeshow (2000), we express the base logistic model with the following equation:

$$\pi \left(x\right)=E(Y|x)=\frac{e^{\beta_{0}+\beta_{1}x_{1}+\ldots +\beta_{k}x_{k}}}{1+e^{\beta_{0}+\beta_{1}x_{1}+\ldots +\beta_{k}x_{k}}}$$

In this equation, *Y* is treated as a binary random variable (possible values: 0, 1). We represent the intercept with β_0_. We represent each coefficient of the independent variables with β_1_ through β_*k*_. Since all of the predictors are categorical, we represent the categories using dummy variables. To examine the relative probabilities, we report exponentiated coefficients and the ORs, in some analyses (e.g., Table 4). To enhance model interpretability, we compute AMEs where feasible (Allison, 1999, Mood, 2009). The employment of AME coefficients also reduces potential biase, which might distort comparisons across groups as well as models with different number of predictors. Interpretations of the AME correspond in approximate terms to interpretations of marginal effects obtained by linear probability models.

For the logistic models, we follow the guidelines provided by the Pew Research Center for implementing its customized survey design weights developed to account for their sampling design. Therefore, we use an estimator relying on a quasi-likelihood approach to logistic regression that provides weighted linearized standard errors appropriate to Pew’s sampling strategy (Archer & Lemeshow, 2006). This approach provides unbiased estimates when used in conjunction with a complex survey sampling design. Since this estimator does not compute the standard chi-square statistics obtained by the maximum likelihood estimator (Hosmer & Lemeshow, 2000), we rely on a specially developed means-residual *F*-test to calculate goodness of fit statistics for each model (Archer & Lemeshow, 2006).

### Modeling Results: Models With all Controls and Both Conditioning Variables

To begin the modeling, we first run a series of four incrementally specified models on the pooled data with all covariates, both controls and conditioning variables. In this way, we ascertain the direction and strength of the effects of videoconferencing and instant messaging on respondents’ future WFH preferences, adjusted for the included covariates. In the initial regression table (Table 2), we see that videoconferencing frequency is associated to a statistically significant degree with WFH preferences in some of the models, while IM frequency is associated with the outcome across all model specifications to a statistically discernable degree. The effect of IM frequency is thus both stronger and more robust to the introduction of control and potential mediating variables than the effect of videoconferencing frequency.

**Table 2.** Average Marginal Effects From Survey-Weighted Binomial Logits [Pooled Sample].

Model	(1)	(2)	(3)	(4)
Specification	Base	Sociodemographics + occupational	Pre-pandemic WFH	Interruptions
VID frequency: often	−0.11*** **(0.03)**	−0.08* **(0.03)**	**−0.05** **(0.03)**	**−0.05** **(0.03)**
IM frequency: often	0.13*** **(0.03)**	0.11*** **(0.03)**	0.11** **(0.03)**	0.09** **(0.03)**
Female		0.05^+^ (0.03)	0.05^+^ (0.03)	0.05^+^ (0.03)
Black non-Hispanic		0.06 (0.05)	0.05 (0.05)	0.03 (0.05)
Hispanic		−0.01 (0.06)	−0.04 (0.05)	−0.03 (0.05)
Other race/ethnicity		0.13^+^ (0.07)	0.12^+^ (0.07)	0.11^+^ (0.07)
Asian non-Hispanic		−0.05 (0.05)	−0.04 (0.05)	−0.04 (0.05)
Age: 30–49 years		0.02 (0.05)	−0.00 (0.05)	−0.02 (0.05)
Age: 50–64 years		0.06 (0.05)	0.04 (0.05)	0.01 (0.05)
Age: 65+ years		0.07 (0.06)	0.02 (0.06)	−0.02 (0.06)
Children in HH		−0.02 (0.03)	−0.03 (0.03)	0.01 (0.03)
College graduate+		−0.06 (0.07)	−0.04 (0.07)	−0.02 (0.06)
Some college		0.03 (0.08)	0.04 (0.07)	0.06 (0.07)
Supervisor		−0.06^+^ (0.03)	−0.06^+^ (0.03)	−0.05 (0.03)
Nonprofit		−0.11** (0.04)	−0.10** (0.04)	−0.09* (0.04)
Government		−0.17*** (0.04)	−0.12*** (0.04)	−0.12*** (0.04)
Self-employed		0.09^+^ (0.05)	−0.01 (0.06)	−0.02 (0.06)
Other		−0.19^+^ (0.11)	−0.20 (0.12)	−0.20 (0.12)
Part-time worker		−0.04 (0.05)	−0.10* (0.05)	−0.08^+^ (0.05)
Pre-pandemic WFH			0.34*** (0.04)	0.35*** (0.04)
More interruptions				−0.14*** (0.030)
Observations	2,500	2,443	2,442	2,437
F	10.879	4.250	7.842	7.903
P	0.000	0.000	0.000	0.000
Svy-F	0.014	1.296	1.704	1.337
Svy-P	1.000	0.233	0.083	0.212

*Note*. Survey corrected SEs. Outcome = wants to work from home (WFH) more often in future; Base = only explanatory variables; WFH = work-from-home.

Bold = focal independent variables

* *p* < .05. **p < .01. ***p < .001. ^+^*p* < .10.

In the base model with only the focal predictors, respondents who engage in greater frequency of instant messaging at the time of the survey were roughly 13% (AME = 0.13, *p* < .001, Model 1) more likely to prefer more frequent WFH in the post-pandemic future, as compared to their counterparts who used IM less intensively. The direction and strength of the association between IM frequency and WFH preferences weakens only to a small degree with the addition of the suite of control variables (models 2–4). After adjusting the model for sociodemographic, work, and internet characteristics, more IM-intensive respondents have an increased probability of 11% to prefer more frequent future WFH (AME = 0.1, *p* < .01, Model 2). The addition of both pre-pandemic WFH frequency and the attentional environment as operationalized by the frequency of interruptions dampen the effect to a small degree, but does not alter its overall magnitude or directionality.

The frequency of videoconferencing displays a slightly different pattern across the four models. Where this technology variable is concerned, the effect starts out as a statistically significant negative association in the base model (AME = −0.11). However, after adjustment for the specified individual-level control variables included in the sociodemographic, occupational, and Internet blocks, the effect shrinks to −0.076, slightly above the threshold for statistical significance (AME = −0.076, *p* < .05, Model 2). Further reductions in the effect of videoconferencing frequency surface with the incorporation of past WFH practices and the attentional environment, as represented by membership in the “more interruptions” category. These controls weaken the effect beyond the threshold of statistical significance.

### Modeling Results: Models With Subsamples Split by Pre-pandemic WFH

**Figure 1.**
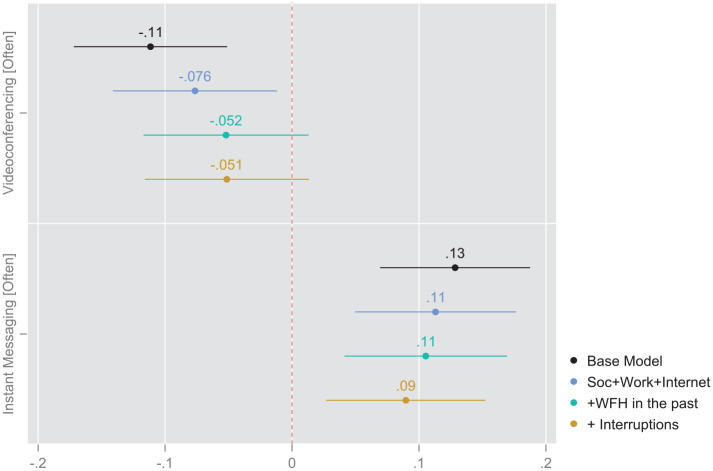
Coefficient plot: Average marginal effects from survey-weighted binomial logits. Based on regression Table 2 (pooled sample).

**Figure 2.**
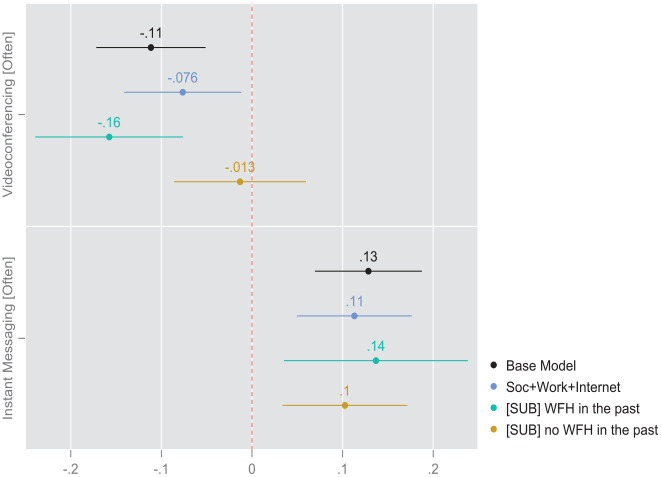
Coefficient plot based on average marginal effects from survey-weighted binomial logits. Based on regression Table 3 (split sample).

It is plausible to claim that the WFH taking place during the pandemic differs significantly from the “enforced” WFH which took place before WFH mandates were implemented by governments worldwide (Evans et al., 2021). Therefore, we expect our primary associations between communication modalities and WFH intentions to vary when those “WFH-experienced” respondents who worked from home before the pandemic as compared to those “WFH-naive” individuals who did not WFH prior to the pandemic. We examine this interaction by running separate analyses (available upon request). We follow our first series of models with pooled data with models estimated on samples split according to the first of the potential confounding variable, namely pre-pandemic WFH. For these split sample models, we present only the coefficients for the two focal technology variables rather than the coefficients for the full set of covariates. From the first regression presented in Table 3 and Figure 1 we discern that, within the subgroup which has some or substantial experience working from home before the pandemic, the frequency of IM and videoconferencing exhibit symmetric but opposite effects on respondents’ WFH preferences. Respondents who used instant messaging more intensively at the time of the survey were roughly 14% (AME = 0.14, *p* < .01, Model 3) more likely to prefer more frequent WFH in the post-pandemic future, as compared to their counterparts who used IM less intensively. Within this same subgroup, the opposite effect was visible among those respondents who used videoconferencing more intensively. Respondents who used instant messaging more intensively at the time of the survey were roughly 16% (AME = − .16, *p* < .001, Model 3) less likely to prefer more frequent WFH in the post-pandemic future, as compared to their counterparts who used IM less intensively. A different pattern emerged among the members of the subgroup of respondents who had not engaged in frequent WFH prior to the pandemic. The impacts of both communication modalities were muted among the respondents who had begun engaging in relatively frequent WFH practices only after the pandemic mandates took effect. This attenuation is particularly pronounced where videoconferencing is concerned. In this case the effect becomes too weak to register as statistically significant (Figure 2).

**Table 3.** Average Marginal Effects From Survey-Weighted Binomial Logits (Focal Predictors Only). Sample Split by Pre-Pandemic WFH Frequency.

Model	(1)	(2)	(3)	(4)
Specification	Base	Full	WFH-past	No WFH-past
VID frequency: often	−0.11*** [−0.17,−0.05]	−0.08* [−0.14,−0.01]	−0.16*** [−0.24,−0.08]	−0.01 [−0.09,0.06]
IM frequency: often	0.13*** [0.07,0.19]	0.11*** [0.05,0.18]	0.14** [0.04,0.24]	0.10** [0.03,0.17]
*N*	2,500	2,443	545	1,897
F	10.879	4.250	1.776	3.601
P	0.000	0.000	0.020	0.000
Svy-F	0.014	1.296	10.712	0.578
Svy-P	1.000	0.233	0.000	0.816

*Note*. Survey corrected SEs. AME = average change in probability of wanting to work from home (WFH) more often in the future; Base = only explanatory variables; Full = all controls included; WFH = work-from-home.

* *p* < .05. **p < .01. ***p < .001.

### Modeling Results: Models With Subsamples Split by Interruptions

**Figure 3.**
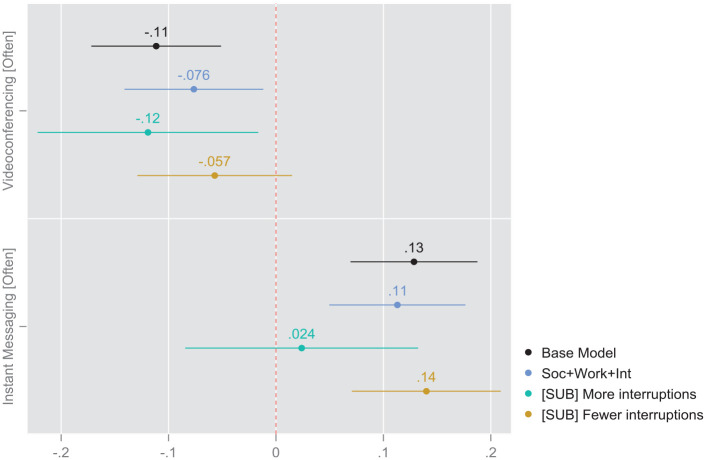
Coefficient plot based on average marginal effects from survey-weighted binomial. Sample split by interruptions, based on regression Table 4 (split sample).

The next set of models estimates the effects of the two focal independent variables on the outcome by splitting the sample according to our primary potential mediating variable, namely the degree of interruptions characterizing the attentional environment of the respondents. The results from this regression model presented in Table 4 and Figure 3 indicate that the effect of instant messaging frequency on the preferred frequency of future WFH is strongly mediated by the degree of interruptions in the WFH environment at the time of the survey, such that the consequences of greater IM frequencies were much more visible for respondents working in less disrupted attention environments (AME = 0.14, *p* < .001, Model 4). However, videoconferencing had a weaker inverse effect, which was only statistically discernable for respondents working in more interrupted environments (AME = −0.12, *p* < .05, Model 3).

**Table 4.** Average Marginal Effects From Survey-Weighted Binomial Logits (Focal Predictors Only). Sample Split by Interruptions.

Model	(1)	(2)	(3)	(4)
Specification	Base	Full	More interruptions	Fewer interruptions
VID often	−0.11*** [−0.17,−0.05]	−0.08* [−0.14,−0.01]	−0.12* [−0.22,−0.02]	−0.06 [−0.13,0.02]
IM often	0.13*** [0.07,0.19]	0.11*** [0.05,0.18]	0.02 [−0.08,0.13]	0.14*** [0.07,0.21]
Observations	2,500	2,443	735	1,703
F	10.879	4.250	2.782	3.582
P	0.000	0.000	0.000	0.000
Svy-F	0.014	1.296	0.575	0.564
Svy-P	1.000	0.233	0.818	0.827

*Note*. Survey corrected SEs. AME = average change in probability of wanting to work from home (WFH) more often in the future; Base = only explanatory variables; Full = all controls included.

* *p* < .05. **p < .01. ***p < .001. ^+^*p* < .10.

## Modeling Results 4: Models With Interaction Terms in Pooled Sample

To confirm and corroborate the results from the prior models derived from split samples, we also ran a full interaction model containing our focal theoretical variables in combination with both of the specified background variables, namely pre-pandemic WFH and the level of interruptions experienced in the respondent’s WFH environment.

The final set of models which we present include interaction terms for both focal independent variables and both background variables in the pooled sample. These results are consistent with the estimates generated in the split models. In the condensed table (Table 5) we present the OR coefficients for the main effects as well as the two-by-two interactions between the two focal theoretical variables and the two focal background variables to highlight to what extent the contributions of these two variables makes any difference to their impact on the preferred future WFH frequency, our dependent variable. As the table shows, with all sociodemographic, work-related, and Internet use controls, the main effect of IM frequency persists, while the main effect of VID is rendered statistically insignificant. As in the split models, two of the four possible interaction effects exhibit statistical significance, namely IM frequency interacted with interruptions (*OR* = 0.49, *p* < .05, Model 2) and videoconferencing frequency interacted with pre-pandemic WFH frequency (*OR* = 0.35, *p* < .05, Model 3).

**Table 5.** Odds Ratios From Survey-Weighted Binomial Logits [Pooled Sample]. (Focal Predictors and Interaction Effects Only).

Model	(1)	(2)
Specification	Base	Full model with interactions
IM often	1.73*** (0.23)	1.89*** (0.34)
VID often	0.62*** (0.08)	0.99 (0.18)
IM often # more interruptions		**0.49** * **(0.15)**
IM often # past WFH most of the time		1.51 (0.72)
VID often # more interruptions		0.73 (0.22)
VID often # past WFH most of the time		**0.35** * **(0.15)**
Observations	2,500	2,437
F	10.879	7.269
P	0.000	0.000
Svy-F	0.014	0.804
Svy-P	1.000	0.613

*Note*. Survey corrected SEs. Base = only explanatory variables; Full = all controls included; Odds Ratios: odds of wanting to work from home more often in the future as compared with baseline odds; WFH = work-from-home.

* *p* < .05. ** *p* < .01. ***p < .001. ^+^*p* < .10.

**Figure 4.**
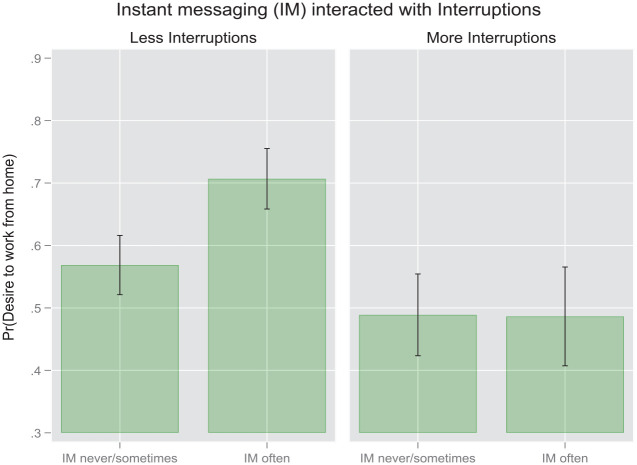
Margin plot for IM interacted with interruptions based on odds ratios from survey-weighted binomial logits [pooled sample].

**Figure 5.**
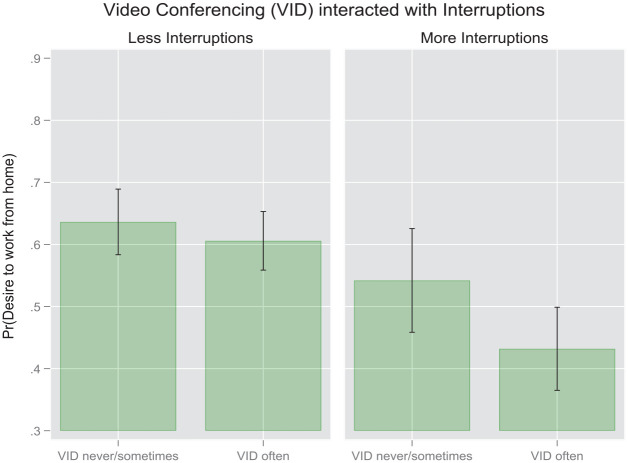
Margin plot for videoconferencing interacted with interruptions based on odds ratios from survey-weighted binomial logits [pooled sample].

These findings are represented visually in the following pair of margin plots (Figures 4 and 5) based on the results in Table 4. As evident from the first plot, the difference in the probability of wanting to work more frequently from home between high-frequency IM users and low-frequency IM users is discernable statistically only for the respondents experiencing fewer interruptions in their WFH environment. However, no parallel difference appears among respondents who endure more interruptions. In the second pair of plots we see that no statistically significant interactions emerge with regard to videoconferencing within either the low-interruption or the high-interruption group.

**Figure 6.**
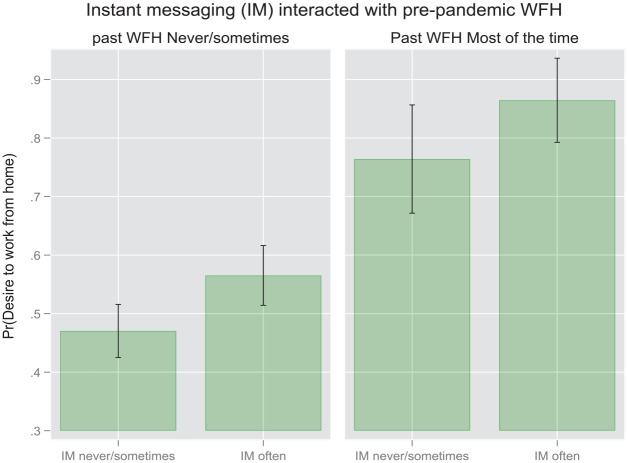
Margin plot for videoconferencing interacted with interruptions based on odds ratios from survey-weighted binomial logits [pooled sample].

**Figure 7.**
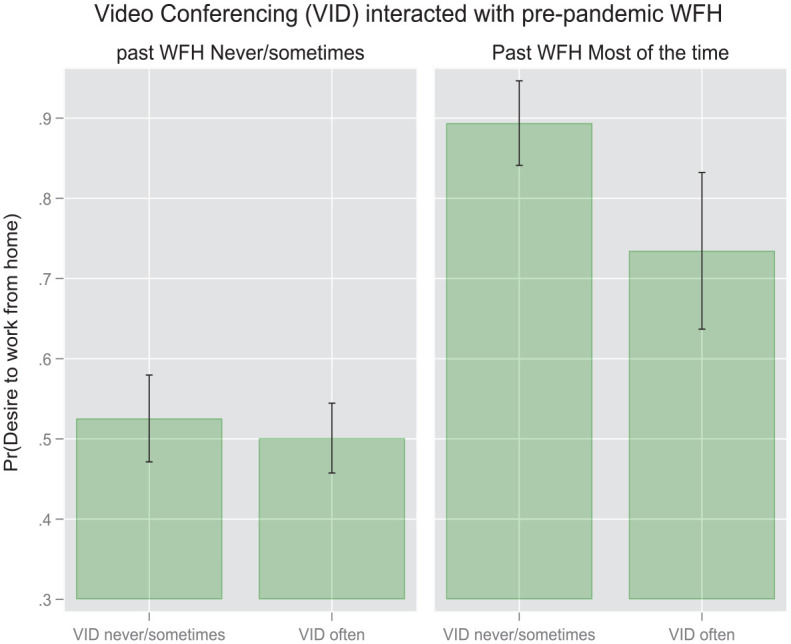
Margin plot for videoconferencing interacted with interruptions based on odds ratios from survey-weighted binomial logits [pooled sample].

In the second pair of margin plots (Figures 6 and 7) we see that the only statistically significant interaction effect materializes with respect to videoconferencing. In this case, the difference in the probability of wanting to work more frequently from home between high-frequency videoconferencers and low-frequency videoconferencers is discernable statistically only for the respondents who worked from home more frequently before the pandemic. No parallel pattern appears with respect to instant messaging. Thus, for those respondents who had already worked from home substantially before the pandemic, less videoconferencing is associated with a higher probability of wanting to work more frequently from home in the future. More experience with pre-pandemic WFH thus intensifies the negative effect of videoconferencing frequency on WFH intentions.

## Limitations

These results cannot be considered definitive for a number of reasons. First, as with all cross-sectional survey-derived data, confounding due to unobserved background variables cannot be ruled out. Second, there are several known potential confounding factors which the data does not allow us to address. Some of the most important of these observable potential confounders are geographic location, pre-pandemic commuting patterns, and types of employer, all of which have been shown to shape WFH practices and could therefore impact WFH intentions as well. According to the ongoing panel study conducted by Barrero, Bloom, and their collaborators on WFH practices in the United States since the start of the pandemic, for instance, both the geographic location and commuting habits of working adults emerge as key factors responsible for the WFH practices of employed adults (Barrero et al., 2022). Taking into account other sources of variation, workers located in U.S. coastal cities with longer average commute times have spent higher proportions of their workweeks working at home rather than at the office, as contrasted with their peers residing outside of coastal areas, outside of cities in general, and facing less adverse commuting conditions. With a more comprehensive dataset, we would be in a position to investigate the role of these factors in conjunction with the technological factors which has been the focus of the study. In addition, it would be ideal to have data pertaining to individuals’ overall work experience, both during the pandemic and prior to the pandemic. Self-reported levels of perceived conflict with co-workers and managers, for example, might bear on the effects of communication modalities on WFH intentions. Finally, the results yielded by these models may well reflect the specific timing of the data collection, in the early days of the pandemic in October of 2020, when WFH was relatively unfamiliar to many workers. Future research should therefore attempt to ascertain whether the differing effects of instant messaging and videoconferencing have persisted or changed during the last 2 years of the pandemic.

## Discussion and Conclusions

The results from the models implemented in this study make a strong case that preferences for future WFH arrangements do not derive solely from the non-technological determinants highlighted in prior studies, whether in psychology, communications, or sociology. Rather, intentions and preferences for WFH stem from the interplay between a variety of non-technological determinants and the affordances of the specific digital communication platforms which have diffused ever more widely during the pandemic. More specifically, as evident in the findings, higher-frequencies of instant messaging correlate with more favorable stances high-frequency future WFH, independent of many other factors associated with WFH intentions. This instant messaging effect is sizeable and noticeable across a range of model specifications. The instant messaging effect is particularly pronounced for individuals who are able to take advantage of low-interruptions WFH environments which impose few non-work demands on their attention. Indeed, the extent to which IM frequency makes frequent future WFH more appealing is heavily mediated by the worker’s attentional environment. This pattern can be interpreted readily through the lens of the existing social-psychological literature. When workers succeed in using IM to communicate with co-workers and managers in an interruption-free environment, they adopt a more favorable stance toward WFH, presumably in part because they can use the modality autonomously and flexibly.

The models show that videoconferencing frequency has a negative effect on WFH intentions, opposite to instant messaging frequency. This effect is weaker than that of IM frequency, and is only statistically discernable in the base model and some of the split models. In these models, higher intensities of videoconferencing predict preferences for lower frequencies of future WFH. The depressing effect of high-intensity videoconferencing is observable most clearly within the subset of individuals who had already worked from home prior to the pandemic, suggesting that intensive videconferencers were particularly disinclined to WFH frequently in the future when they were already familiar with WFH constraints due to their pre-pandemic WFH experience. Indeed, within the small subgroup of workers who had worked from home before the pandemic the most frequently, never having videconferenced raised the odds of wanting to WFH more frequently in the future by a statistically significant extent. Lower probabilities of preferring high-frequency WFH were also statistically discernable among the intensive videoconferencers who worked in high-interruption environments, an intuitively plausible pattern which reflects the intuitively understandable frustrations of videoconferencing in setting replete with visual and auditory distractions.

Both the findings with respect to instant messaging and videoconferencing can be understood in light of the social science literature on communication modalities and work along multiple analytic dimensions. The affordances of high-frequency instant messaging—primarily its temporal flexibility as medium of communication which can be more or less synchronous, depending on the way in which it is used—appear to enhance its appeal for workers, thereby making high-frequency WFH more attractive as a future option. The ease of multitasking also makes text messaging platforms like Slack less intrusive and potentially stress-generating than videoconferencing, which demands a greater share of the worker’s attention. Clearly, the combination of the “lean” qualities of text messaging in addition to its “multichronic” character makes it more pleasant and manageable across a broad range of workers and workplaces than videoconferencing, a positive effect which is amplified in low-interruption environments. Because the positive impact of IM is largely due to the “sense of copresence” (Darics, 2014, p. 352) workers are able to generate through autonomously initiated communication, it follows that distractions in their WFH environment would erode this sense of copresence and therefore lessen the appeal of WFH. Conversely, videoconferencing imposes heavier attentional and presentational demands on participants as compared to instant messaging; participants must coordinate meeting times, stage their own appearance, manage the setting, and block out potential distractions.

However, the question remains open whether the observed differences in WFH intentions associated with technology usage are due wholly or partially to the affordances of the technology. For instance, the differential effect of these two communication modalities on WFH intentions might be a function of differences in the degree of conflict or stress across workplaces, industries, or types of work team. For example, it may well be the case that high-frequency instant messaging is a feature of workplaces and work teams characterized by less conflict and friction than high-frequency videoconferencing, a possibility well worth exploring in future research. Such a difference may reinforce differences due to the technological affordances of these two communication modalities in ways both expected and unexpected.
